# Immunological Interplay at the Ocular Surface of Stevens-Johnson Syndrome, Ocular Cicatricial Pemphigoid, and Ocular Graft Versus Host Disease

**DOI:** 10.1167/iovs.67.6.26

**Published:** 2026-06-15

**Authors:** Tejaswini Pingali, Swaminathan Sethu, Sukesh Kommana, Swati Singh, Swapna S. Shanbhag, Arkasubhra Ghosh, Sayan Basu, Vivek Singh

**Affiliations:** 1 Centre for Ocular Regeneration (CORE), Professor Brien Holden Eye Research Centre, L. V. Prasad Eye Institute, Hyderabad, India; 2 Manipal Academy of Higher Education (MAHE), Manipal, India; 3 GROW Research Laboratory, Narayana Netralaya Foundation, Bangalore, India; 4 Shantilal Shanghvi Cornea Institute, L. V. Prasad Eye Institute, Hyderabad, Telangana, India

**Keywords:** Stevens-Johnson syndrome (SJS), ocular cicatricial pemphigoid (OCP), ocular graft versus host disease (oGVHD), chronic inflammation, ocular inflammation, Autoimmunity

## Abstract

Rare ocular surface inflammatory disorders (OSIDs), such as ocular cicatricial pemphigoid (OCP; 1.3–2/million), Stevens-Johnson Syndrome (SJS; 1–5/million), and ocular graft versus host disease (oGVHD; occurring in >50% of chronic GVHD cases) share overlapping clinical phenotypes. Their defining features include conjunctival cicatrization and chronic inflammation. An incomplete understanding of the underlying immunopathogenesis of these conditions has hindered the development of targeted therapies, leaving patients at risk of permanent vision loss and highlighting the need to define distinct ocular immune profiles. Existing literature indicates elevated levels of neutrophils and macrophages/monocytes, along with major increases in their related secretory factors, IL-8 (*P* < 0.05) and TNF-α (*P* < 0.05), at the ocular surface across all three groups. Whereas both B cells and cytotoxic T cells are elevated in oGVHD, OCP is characterized by elevated B cells and a concomitant reduction in cytotoxic T cells. Apart from neutrophils and macrophages/monocytes, the local immune landscape—especially in SJS—has been underexplored. At the molecular level, extensive studies of tears have reported elevated levels of IL-17, CXCR1 (*P* < 0.01), CTGF (*P* < 0.05) in OCP; IFN-α and IFN-γ (*P* < 0.05) in oGVHD; and MCP-1, MIP-1β (*P* < 0.05) in SJS. These studies also indicate that sampling methods and patients’ medication status may influence immune profile outcomes. Comprehensive immunophenotyping studies, coupled with the establishment of pathological links to respective molecular profiles, could advance understanding of condition-specific disease biology.

Ocular surface inflammatory disorders (OSIDs) comprise a heterogenous group of conditions arising from distinct etiologies but share severe ocular inflammation as a common clinical feature. This review focuses on three OSIDs – Stevens-Johnson syndrome (SJS), ocular cicatricial pemphigoid (OCP), and ocular graft versus host disease (oGVHD)—all of which share the pathological hallmark of progressive, chronic conjunctival cicatrization. Despite their low global annual incidence rate (approximately 1–5 cases/million),^1^^–^^3^ standard management^3^—namely, broad-spectrum immunosuppressants and repeated surgeries—has largely been ineffective in resolving ocular immune infiltration and often results in permanent vision impairment. To overcome these clinical challenges, it is critical to understand and compare the underlying immune mechanisms at the ocular surface that are precipitated by these OSIDs.

Current insights into disease pathogenesis classify SJS as a type IV hypersensitive reaction,^4^^,^^5^ OCP as a type II hypersensitive reaction,^1^^,^^6^^–^^8^ and oGVHD as an alloimmune disorder.^3^ In SJS, exposure to specific drugs trigger severe ocular surface damage, with eyelid margin keratinization as a distinct clinical feature.^2^^,^^4^^,^^5^ Keratinized eyelids create a sandpaper-like effect making blinking extremely painful and ultimately compromises vision. In OCP, auto-reactive B cells produce antibodies against the conjunctival basement membrane proteins, clinically manifesting as symblepharon formation and conjunctival fibrosis.^1^^,^^6^^–^^8^ The oGVHD, unique to individuals undergoing hematopoietic stem cell transplantation, is characterized by impaired tear production, commonly attributed to elevated T-cell infiltration of the lacrimal gland.^3^^,^^9^^,^^10^ Notably, the ocular manifestations observed in these OSIDs are thought to represent sequelae of an initial systemic immune perturbation. Systemically, the role of auto-reactive B cells,^1^^,^^6^^–^^8^ cytotoxic T cells,^2^^,^^4^^,^^5^ and helper T cells^3^^,^^9^^,^^10^ have been well characterized in OCP, SJS, and GVHD, respectively. However, the key immune mediators responsible for disrupting ocular surface immune homeostasis causing the tissue damage that culminates in permanent vision loss remains poorly defined across these OSIDs.

In this context, this narrative review aims to evaluate and compare the reported cellular and molecular immune dysregulation at the ocular surface in patients clinically diagnosed with SJS, OCP, and oGVHD. Additionally, we discuss existing challenges and highlight future perspectives to enhance the development of targeted and disease-specific management strategies.

## Ocular Surface Immune Homeostasis

The eye is the primary organ for vision in our body. Its location and function make it a unique threshold for endogenous and exogenous threats, like internal stressors and pathogens. The inability of various ocular cells (corneal endothelium and neurosensory retina) to regenerate makes the visual axis extremely vulnerable to chronic inflammation inevitably leading to vision loss.^11^ Evolutionarily, the eye has been proposed as an immune-privileged site, characterized by its ability to prolong the survival of allogenic tissue that would typically undergo rapid rejection in other sites. This phenomenon was primarily demonstrated in 1900s by Sir Peter Brian Medawar, who placed a foreign tissue in the anterior chamber of a mouse's eye and observed no signs of graft rejection.^12^ Initially, immune privilege was considered to be the site's inability to elicit an immune response against a specific antigen. This was corrected by Kaplan and Streilein, who demonstrated a specialized, systemic immune response in mice with an allogenic graft transplanted to the anterior chamber of an eye.^13^

Over the years, the eye's immune privilege has been vividly debated with increasing evidence suggesting it to be more of an “immune deviation” process. According to this concept, an active interplay exists between blood-tissue barriers (the blood-aqueous and blood-retinal barriers) and defined regulators in the eye to maintain its distinct immunological milieu. These immune regulators include the macrophage migration inhibitory factor (MIF), which is majorly present in the aqueous humor and mitigates natural killer (NK) cell-mediated, nonspecific lysis in the eye.^14^^,^^15^ The aqueous humor is additionally enriched with neuropeptides (vasoactive intestinal peptide and somatostatin) and α-melanocyte stimulating hormone that suppress nonspecific T-cell activation and promote T regulatory cells production to alleviate inflammation.^15^ Corneal endothelial cells are reported to have cell surface molecules like CD95L to promote T cell apoptosis thereby increasing graft survival rate.^14^ Corneal cells additionally express vascular growth factor receptors and Major Histocompatibility Complex (MHC)-I/II molecules which enhance ocular surface homeostasis.^16^

However, in case of a breach, ocular cells, especially in the anterior segment, send signals to the spleen/thymus and recruit immune cells/factors to minimize nonspecific tissue damage – the Anterior Chamber Associated Immune Deviation (ACAID).^15^ Briefly, ACAID is a cascade of immune reactions which activates resident ocular antigen presenting cells (APCs) like CD11b+CD11c– macrophages/monocytes, CD11c+CD11b–/CD207low non-Langerhans dendritic cells (DCs), CD11c+CD11b+CD207+ Langerhans DCs.^15^^,^^17^ Activated APCs enter the spleen, secrete MIF and CCL5 to recruit Natural Killer T (NKT) cells and generate suppressor regulatory T (Treg) cells (CD4+/CD8+).^15^ These regulatory T cells aid in reducing the levels of IFN-γ and IL-12, thereby preventing nonspecific Th1-driven hypersensitive response. Some ocular APCs through receptor C3b expression, induce production of IL-10 and TGF-β to regulate ocular immune balance.^15^

Researchers have also reported a diversity of immune cells in the healthy human eye. The cornea, the most explored region, has macrophages accounting for 50% of its immune population.^18^ These resident corneal macrophages, based on CCR2 receptor expression, are categorized into CCR2– (M2-like macrophages) and CCR2+ (M1-like macrophages) macrophages.^18^ Another macrophage subgroup is the Langerhans cells which are reported in the limbus, conjunctiva, and cornea.^18^ Human cornea and conjunctiva are also reported to host mast cells whose numbers have been reported to increase after corneal transplantation, signifying their role in graft tissue maintenance. Mast cells also help in maintaining corneal nerve growth and limbal vasculature through the production of distinct neurotrophins and vascular endothelial growth factors, respectively.^18^ Recently, γδ T cells have also been identified in the human conjunctiva and cornea, where they aid in corneal wound repair and regulating inflammation.^18^^,^^19^ The human limbus and bulbar conjunctiva have a reported presence of plasmacytoid dendritic cells, which apart from providing IFN-α mediated anti-viral immunity, also promote Treg cell production.^20^ Other immune cells reported in the conjunctiva are innate lymphoid cells (ILCs), which are categorized as NK cells, possessing an IFNγ-mediated cytotoxic effect through Th-17 activation, and non-toxic ILCs whose precise role and location at the ocular surface remains unexplored.^18^

## Ocular Surface Inflammatory Disorders

Under normal conditions, multiple cellular and molecular factors maintain ocular homeostasis, but triggers like environmental factors, drugs, pathogens, allergens, or genetics can disrupt this balance and initiate a chronic inflammatory cascade at the ocular surface. The prolonged persistence of inflammation in the eye is a key clinical phenotype of OSIDs. The OSID is an umbrella term to describe all the conditions with origins that share chronic ocular surface inflammation as the common symptom.^21^ These OSIDs are OCP, SJS, and oGVHD, primary Sjögren syndrome, ocular allergic disorders, rosacea, rheumatoid arthritis, and systemic lupus erythematosus. Most of these conditions are autoimmune in nature, and characterized by consistent recruitment of inflammatory mediators to the ocular adnexal regions, including eyelids, cornea, conjunctiva, meibomian gland, and lacrimal gland.^21^ Because ocular adnexa have major tear film producing components, most of these OSIDs have dry eye disease as the most common comorbidity.^21^

The review focuses on understanding the current pathophysiological understanding of the three of the OSID conditions—OCP, SJS, and oGVHD, in terms of etiology, epidemiology, pathogenesis, clinical diagnosis, and ocular surface immune dysregulation by both cellular and molecular factors which have been detailed in the sections below.

### Ocular Cicatricial Pemphigoid

#### Etiology and Epidemiology

**Figure 1.**
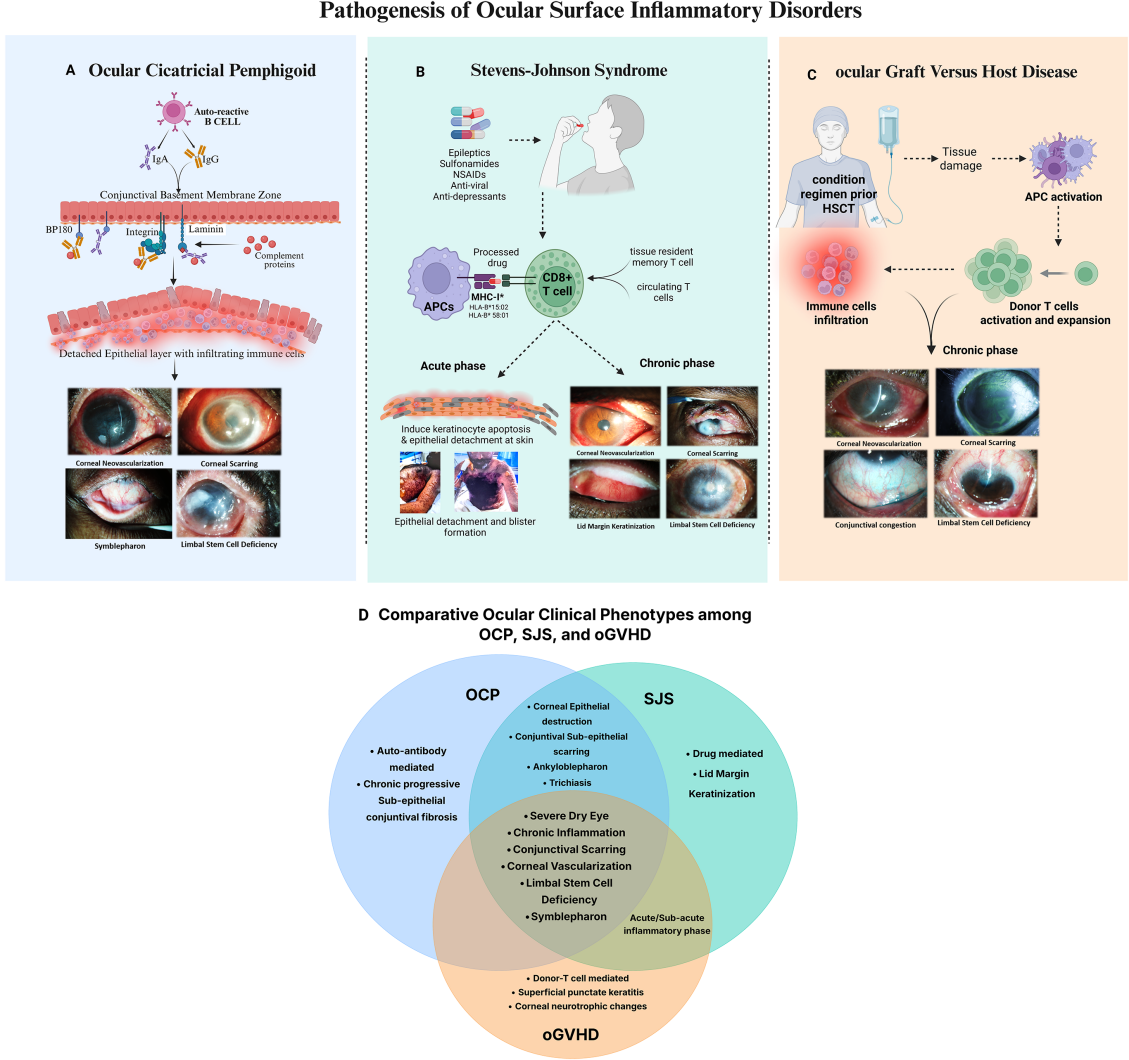
**Clinical pathology and symptoms of the focused ocular surface inflammatory disorders** **.** (**A**) Ocular cicatricial pemphigoid is an autoimmune reaction where B cells lose the immunological tolerance and produce self-attacking antibodies (IgG/IgA) toward proteins like laminins, integrins, and bullous pemphigoid-180 present in the conjunctival basement membrane. This interaction activates complement proteins to initiate an inflammatory cascade characterised by heavy infiltration of immune cells and secretory factors to the ocular surface. The inflammatory milieu causes detachment of the conjunctival epithelial layer from its sub-epithelium, clinically marked as symblepharon formation, conjunctival fibrosis/scarring, corneal neo-vascularisation with severe cases having limbal stem cell deficiency (LSCD). (**B**) Stevens-Johnson syndrome is a delayed type IV hypersensitive reaction triggered by drugs like epileptics, sulphonamides, non-steroidal anti-inflammatory drugs (NSAIDs), anti-virals, and anti-depressants. These drugs bind to restricted MHC-I molecules on the circulating antigen presenting cells (APCs), activating and differentiating CD8+ cytotoxic T cells. These systemically activated CD8+ Tc cells cause keratinocyte death, and epithelial cell detachment (Nikolsky's sign) leading to blister formation at mucocutaneous surfaces. At the ocular level, the reported symptoms include eyelid margin keratinization, corneal/conjunctival scarring, LSCD, and corneal neovascularization. (**C**) Ocular graft versus host disease is restricted to the individuals who undergo allogenic hematopoeitic stem cell transplantation, where exposure to radioactive/chemotherapy initiates host tissue damage. This further activates and alter host tissue specific APCs that present self-antigens to donor T cells. The activated donor T cells initiate chemtotactic movement of other immune cells and cause release of tissue hampering inflammatory factors to the ocular surface. Ocular clinical phenotypes include corneal neovascularization, corneal scarring, LSCD, congestion of conjunctiva, with severe ocular surface inflammation. Clinical images for all three conditions were obtained from the records of patients who underwent ocular examination at L. V. Prasad Eye Institute, Hyderabad. (**D**) Venn Diagram summarizes common and distinct ocular clinical phenotypes across OCP, SJS, and oGVHD. Based on current understanding, all three OSIDs share severe dry eye, chronic inflammation, conjunctival scarring, symbelepharon formation, corneal vascularization, and LSCD as the top common clinical manifestations. The schematic diagram was created in https://BioRender.com.

OCP belongs to the mucus membrane pemphigoid group of conditions, which are autoimmune in nature, primarily affecting the subepithelial/epidermal surfaces of the skin and mucosa.^6^ It has a mean annual incidence rate of 1.3 to 2 per million and average age of onset between 60 and 65 years.^1^ With loss of peripheral tolerance, auto-antibodies (IgG/IgA) are generated against the conjunctival basement membrane proteins, such as bullous pemphigoid protein 180, laminin 332, and integrins^1^^,^^7^ (Fig. 1A). Recent research suggests the existence of a genetic predisposition (HLA-DQB1*03(01), DRB1*04, and HLA-B12 alleles) in OCP.^6^

#### Pathogenesis

OCP is categorized as a type II hypersensitive reaction, where auto-antibodies activate complement proteins (C3/C5) and initiate an inflammatory cascade at the ocular surface.^8^ This immune reaction leads to a separation of the epithelial membrane from the subepithelial layers of conjunctiva resulting in blister formation and tissue scarring (see Fig. 1A).^1^^,^^6^^–^^8^

#### Clinical Diagnosis

The current gold standard diagnosis for OCP includes the direct immunofluorescence-based approach with a sensitivity of 30% to 80% where auto-antibodies (IgG and IgA) and complement proteins are detected in the conjunctival basement zone.^7^^,^^22^ Clinical signs include symblepharon formation, conjunctival blistering, corneal scarring, corneal neovascularization, and ankyloblepharon formation (see Fig. 1A).^6^^,^^7^ Mondino-Brown and Tauber classifications are the most commonly adopted methods to grade the disease severity.^23^

#### Ocular Surface Immune Dysregulation Reported At

##### Cellular Level

**Figure 2.**
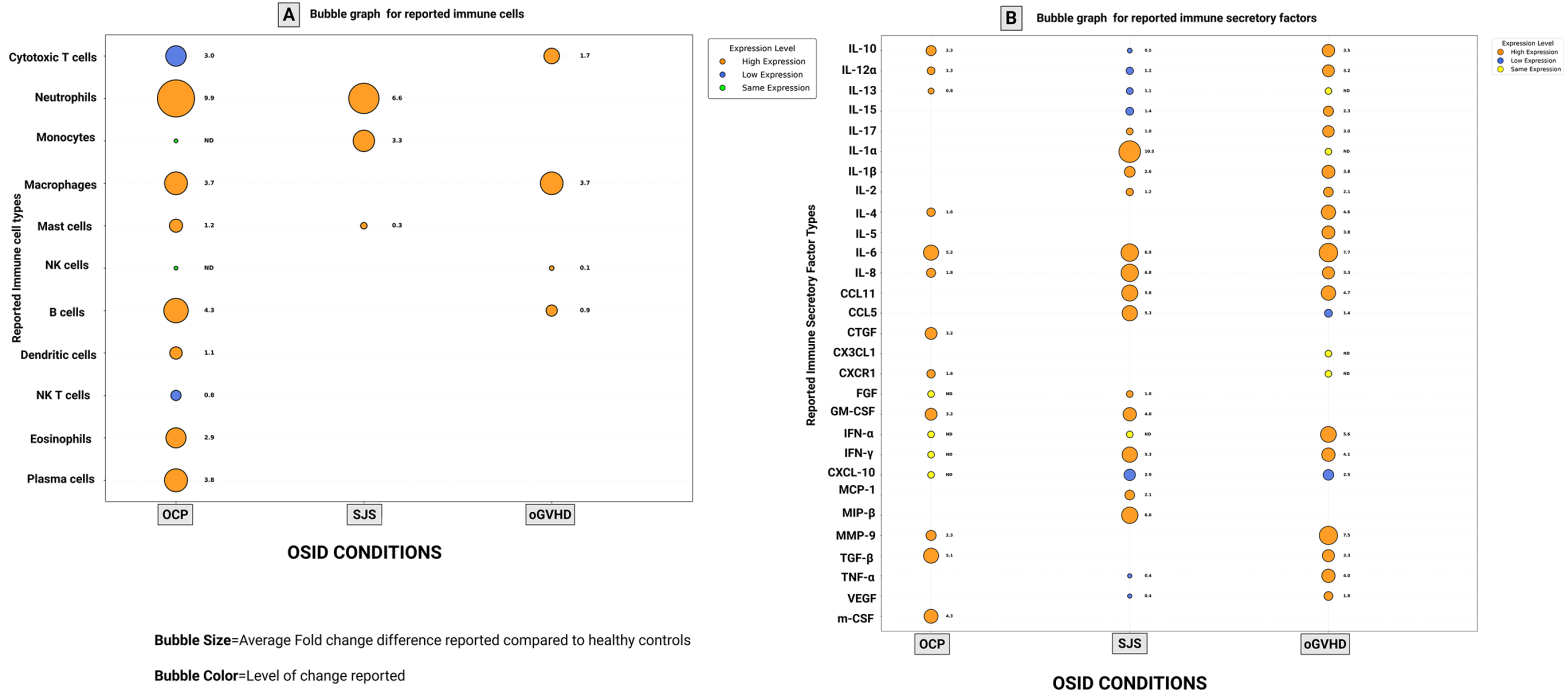
**Bubble graphs comparing the common and distinct (A)** **immune cells and (B)** **secretory factors reported at the ocular surface of OCP, SJS, and oGVHD.** All the comparisons reported are with repect to healthy controls. The size of the bubble corresponds to the magnitude of average fold change reported in various studies with respect to that specific cell/factor in each OSID group. These average fold change values have been mentioned adjacent to each bubble, where ND defines “no difference” in fold change reported between disease and healthy control. Further, the color of the bubble corresponds to relative level of change reported with *orange* denoting Higher expression change, *blue* denoting lower expression change, and *green/yellow* denoting no change in expression for the specified immune cell/factor in each OSID group compared with healthy controls. The bubble plot was generated using Python in Jupyter Notebook.

At the conjunctival surface, using ocular surface impression cytology (OSIC), Williams et al. reported elevated neutrophils (no. of patients [*n*] = 57),^24^ and lower cytotoxic T cells (Tc; *n* = 57),^24^ whereas no difference (*n* = 57) was noted in levels of NK cells,^24^ B cells,^24^ and helper T cells (Th), compared to healthy controls (HCs).^24^ Neutrophils were observed to be elevated in both inflamed and non-inflamed eyes of patients with OCP (53% of patients on immunosuppression).^24^ On the other hand, immunohistochemical studies in the conjunctival tissue of patients with OCP, elevated macrophages (*n* = 10),^25^ Tc cells (*n* = 10),^26^ Th cells (*n* = 10),^26^^–^^28^ and B cells (*n* = 6)^27^ were reported, especially in the stromal/substantia propria regions (irrespective of patients’ medication status), compared with HCs. Figure 2A is a bubble graph overview of the reported immune cells at the ocular surface of OCP to date.

##### Molecular Level

IL-6 was studied in most of the papers and was reported to be higher in tears (*n* = 3),^29^ conjunctiva (*n* = 5),^30^ and aqueous humor (*n* = 4),^31^ of patients with OCP, when compared with HCs (irrespective of patients’ medication status). Further higher levels of macrophage-associated factors like macrophage colony-stimulating factor (m-CSF; *n* = 10)^25^ and macrophage MIF (*n* = 10; Razzaque et al., 2004) was observed in OCP conjunctiva, compared with HCs. IL-8/CXCL8, was observed to be elevated (*n* = 4 and 20)^29^^,^^32^ in OCP tears compared with HCs. The substantia propria region of patients with OCP, when compared with HCs, further reported elevated levels of various fibrosis-related factors like IL-13 (both acute [*n* = 10] and chronic [*n* = 20])^33^^,^^34^ and IL-17 (*n* = 4 and 5).^30^ Other factors, like IL-4 (*n* = 10)^35^ and TNF-α (*n* = 8 and 10)^36^^,^^37^ were also reported to be elevated in the conjunctiva of OCP, compared with HCs. Figure 2B is a bubble graph providing an overview of the reported secretory factors at the ocular surface of patients with OCP to date.

### Stevens-Johnson Syndrome

#### Etiology and Epidemiology

Severe cutaneous adverse reactions (SCARs) are a group of life-threatening drug-induced and T cell-mediated hypersensitive reactions. The World Health Organization classifies SCAR into type I/immediate and type IV/delayed type hypersensitive reactions, based on their immunological mechanisms.^4^^,^^5^ SJS and toxic epidermal necrolysis (TEN) are type IV hypersensitive reactions, triggered by drugs, primarily affecting the mucosal surfaces (oral/ocular/genital) in 50% to 90% of cases.^2^ SJS and TEN fall in the same disease spectrum but differ based on the percentage of total body surface area (TBSA) affected, with SJS having <10% TBSA detachment, TEN having >30% TBSA detachment, and SJS-TEN overlap having 10% to 30% TBSA detachment.^38^ Annually, SJS affects one to five individuals/million, with a female preponderance (2:1).^38^ The most common SJS-associated drugs are antibiotics, anti-epileptics, benzodiazepines, diuretics, non-steroidal anti-inflammatory drugs, anti-depressants, anti-neoplastic, and anti-viral agents.^38^ Some of these drugs have distinct genetic predisposition like carbamazepine with HLA-B*57:01 and allopurinol with HLA-B*58:01.^4^

#### Pathogenesis

Being type IV delayed-type hypersensitive reaction, SJS is characterized by drug interaction (directly/indirectly) with restricted MHC antigen and activating Tc cells (circulating/tissue-resident). Systemically, activated Tc cells recruit NK cells, monocytes, and macrophages through Fas/Fas ligand, tumor necrosis factor-related apoptosis-inducing ligand, granulysin, TNF-α, or perforin induce skin keratinocyte apoptosis^4^^,^^5^^,^^38^^,^^39^ (Fig. 1B).

#### Clinical Diagnosis

In the acute stage, patients with SJS present conjunctival and corneal epithelial denudation, eyelid margin epithelial defects, and corneal ulceration. While in the chronic stage, patients have keratinized lid margins, severe dry eye, symblepharon, and limbal stem cell deficiency along with corneal and conjunctival scarring (see Fig. 1B).^2^^,^^4^^,^^39^ Due to the absence of a single, standard diagnostic test, patch/intradermal testing combined with severity-of-illness SCORe for TEN (SCORTEN) grading helps in identifying the culprit drug.^4^^,^^39^

#### Ocular Surface Immune Dysregulation Reported at

##### Cellular Level

**Figure 3.**
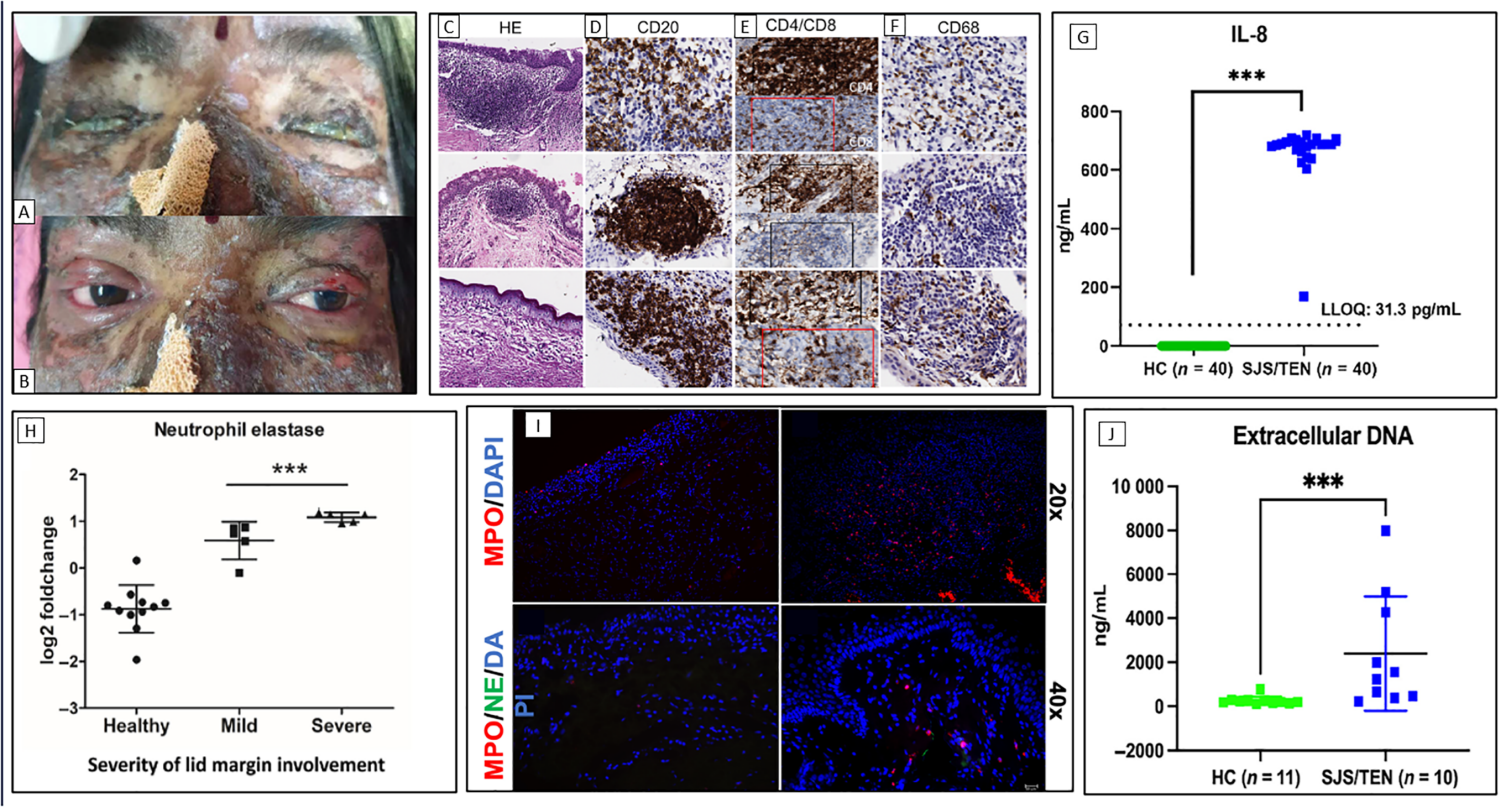
**Immunological changes reported at the ocular surface of chronic SJS.** Our previous study by Singh et al.,^93^ showing (**A,** **B**) clinical phenotypes observed at the chronic stage of the patients with SJS. (**C**) Eyelid margin tissue sections from patients with SJS showing infiltration of immune cells in the sub-epithelial region which were characterized to be positive for markers like (**D**) CD20 (B cell), (**E**) CD4/CD8 (T cell), and (**F**) CD68 (macrophages). Another study by Koduri et al.^41^ showing elevated levels of neutrophil and its associated proteins at the ocular surface including (**G**) IL-8 and (**H**) degranulation proteins like neutrophil elastase. Degranulation of neutrophils was validated through (**I**) positive expression of both neutrophil elastase and myeloperoxidase (MPO) markers in chronic SJS eyelid margin tissues. (**J**) The elevated extracellular DNA content in chronic SJS tears compared to healthy controls suggested neutrophils undergoing netosis. (Copyright permission: Images A–J are adapted and modified from our publications by Singh et al.^93^ and Koduri et al.^41^).

Using OSIC, Williams et al. reported high neutrophil and monocyte levels, at the SJS-affected conjunctival surface (*n* = 10; on immunosuppression), compared with HCs.^40^ Conjunctival neutrophils remained elevated even after 12 months (*n* = 9).^40^ Further, Koduri et al. also reported elevated neutrophils in the keratinized eyelid margin tissues (*n* = 12) of patients with chronic SJS (on immunosuppression) compared with HCs.^41^ Some studies suggest ocular neutrophils in SJS could be undergoing NETosis, due to marked elevation in neutrophil degranulation proteins like neutrophil defensin, neutrophil elastase, and myeloperoxidase (*n* = 12 and 51, and 4; Fig. 3).^41^^–^^43^

Elevated conjunctival monocytes could be correlated with high levels of associated chemokine monocyte chemotactic protein (MCP)-1 in both tears (*n* = 61 and 30) (Yoshikawa et al., 2020^44^; Gurumurthy et al., 2018b^45^) and the aqueous humor (*n* = 2).^31^ Among these studies, patients with SJS in the Gurumurthy et al. (Gurumurthy et al., 2018b^45^) study were not on medication, whereas this information was missing for other studies.^31^ (Gurumurthy et al., 2018b^45^; Yoshikawa et al., 2020^44^).

Another key immune cell reported widely with SJS is the Tc cell whose elevated levels have been reported to play a critical role in the systemic stage of SJS (*n* = 23).^46^ Tc cells through Fas ligand (*n* = 6, 15),^47^^,^^48^ perforins, or granzymes exhibit cytotoxic activity causing keratinocyte apoptosis.^42^ Most of these studies used skin biopsies (*n* = 23),^46^ blister fluids (*n* = 15),^47^ and peripheral blood samples (*n* = 15).^47^ Very few attempts have been made to understand Tc cell status at the ocular level; a single study by Williams et al., using OSIC, observed no difference in Tc levels at the conjunctival surface in patients with chronic SJS (*n* = 10) compared with HCs.^40^

Other immune cells, like NK cells (*n* = 11)^49^ and macrophages (*n* = 6),^48^ have also been reported to be higher in non-ocular samples (skin biopsies), where Schlapbach et al. also showed a positive correlation between NK cells and granulysin.^49^ Thus, it is important to understand their levels at the ocular surface. Figure 2A is a bubble graph overview of the reported immune cells at the ocular surface of SJS to date.

##### Molecular Level

**Figure 4.**
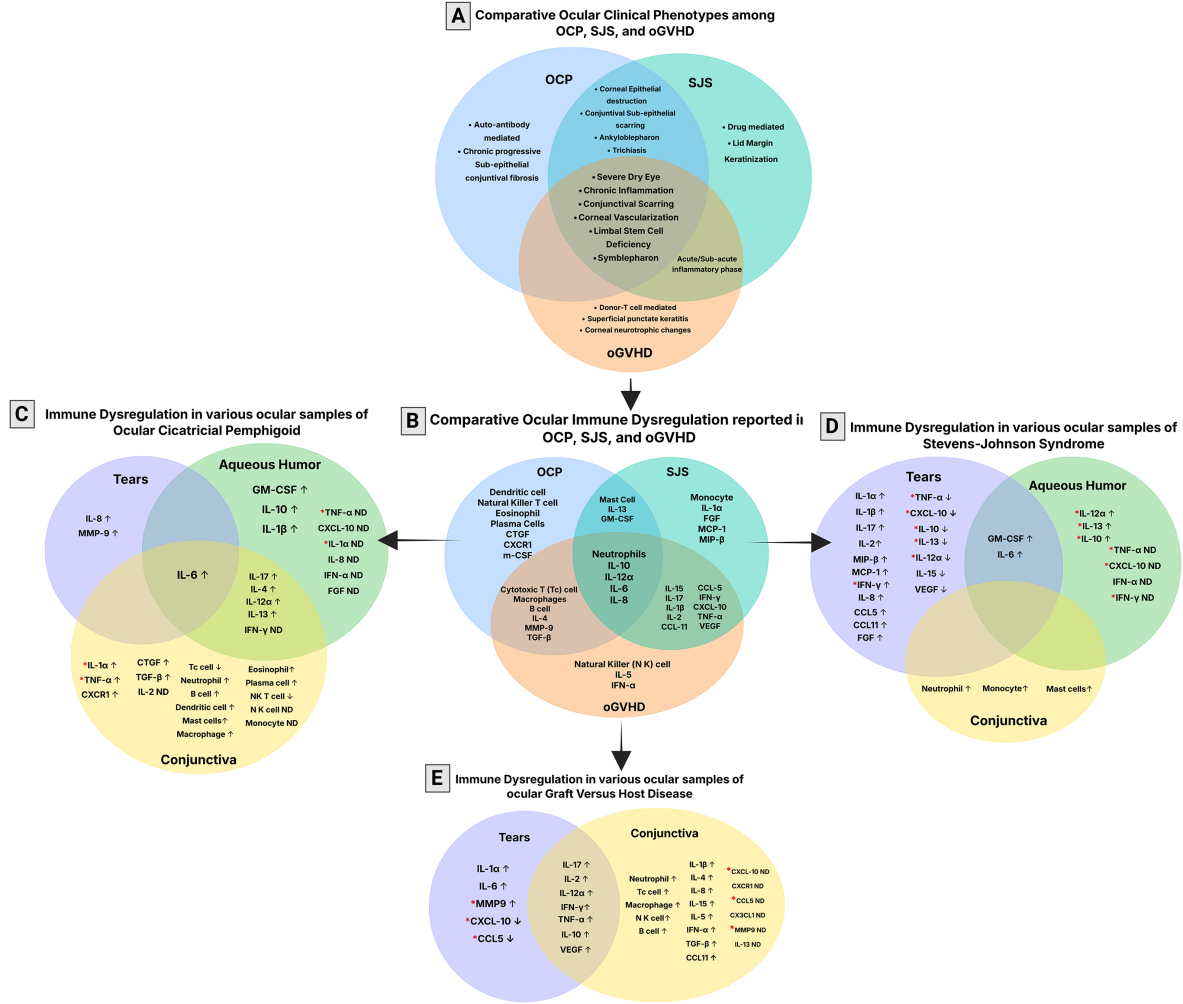
**Venn diagrams summari** **z** **ing the immune dyregulation reported at the ocular surface of OCP, SJS** **,** **and oGVHD and correlating with disease-specific phenotypes.** (**A**) Clinical phenotypes reported distinctly and in common at the ocular surface of the focused OSID groups (OCP, SJS, and oGVHD). These clinical features could be correlated to (**B**) the reported immunological changes (both cellular and molecular) at the ocular surface of these OSID groups. (**C–E**) Further, the reported ocular immune dyregulation could be dissected based on sample type used for immune profiling and has been represented as the venn diagram for **C** ocular cicatricial pemphigoid (OCP), **D** Stevens Johnson syndrome (SJS), and **E** ocular graft versus host disease (oGVHD). The *red star* in **C** to **E** highlights the immune cells/factors whose levels have been observed distictly variant in different ocular samples, underlining the plausible effect of sampling technique on ocular immune profiling.

In tears samples of patients with chronic SJS, various elevated cytokine levels have been reported: IL-1β (*n* = 12 and 25),^44^ (Gurumurthy et al., 2018b) IL-2 (*n* = 25 and 12) (Gurumurthy et al., 2018b),^50^ and IL-8 (*n* = 25, 30, 12, and 61) (Gurumurthy et al., 2018b),^44^^,^^50^^,^^51^ For some secretory factors, like IFN-γ, IL-13, TNF-α, and IL-17, contrasting levels have been observed (Fig. 4E). Studies eliminating the medication effect observed reduced tear IFN-γ (*n* = 25) (Gurumurthy et al., 2018b^45^), whereas studies with unclear patients’ medication status observed both elevated (*n* = 30)^51^ and no difference in IFN-γ levels (*n* = 12 and 61)^44^^,^^50^ in chronic SJS tears compared with HCs. Similarly, IL-13 was higher in aqueous humor (*n* = 2; on immunosuppression),^31^ whereas it was lower in SJS tears (*n* = 30) of patients not on medication (Gurumurthy et al., 2018b). Another cytokine, IL-17, was higher in chronic SJS tears with subjects not on medication (*n* = 5 and 25) (Gurumurthy et al., 2018b),^52^ whereas another study (*n* = 12) with unclear medication status reported no difference in tear IL-17 levels.^50^ A similar discrepancy was observed in tear TNF-α levels with Gurumurthy et al. reporting low TNF-α (n = 25, not on medication) (Gurumurthy et al., 2018b), whereas Koduri et al. (*n* = 12)^50^ and Yoshikawa et al. (*n* = 61),^44^ with unclear medication status, reported no difference in TNF-α levels compared with HCs.

IL-6 and macrophage inflammatory protein (MIP-1α and MIP-1β)^31^ have been observed to be elevated in tears (IL-6; *n* = 12, 30, and 61)^44^^,^^50^^,^^51^ and the aqueous humor of patients with chronic SJS with unclear medication status. Although, IL-6 and MIP are known cytokines mostly associated with B cells and macrophages, however, it is difficult to extrapolate the status of respective cells based on such pleotropic cytokine levels.

In chronic SJS, CCL11 and CXCL10 are two major chemokines studied at the ocular surface. CCL11/Eotaxin-1 was reported higher in SJS tears (*n* = 30 and 61)^44^^,^^51^ compared with HCs, irrespective of medication status. However, lower tear CXCL10 levels have been reported (*n* = 25, 12, and 20) (Gurumurthy et al., 2018; Koduri et al., 2021a; Ueta et al., 2017) in patients who were not on medication. Figure 2B is a bubble graph overview of the reported secretory factors at the ocular surface of SJS to date.

### Ocular Graft Versus Host Disease

#### Etiology and Epidemiology

The oGVHD is a potentially lethal complication observed in individuals undergoing hematopoietic stem cell transplantation or bone marrow transplantation.^3^ To prevent graft rejection, patients generally receive a conditioning regimen with immunosuppressive agents before transplantation. This condition regimen step not only suppresses host immunity but also damages various organs, including oral mucosa, eyes, skin, gastrointestinal tract, liver, and genitalia.^3^ Ocular involvement has been reported less in (<10%) acute GVHD cases and more (30%–80%) in chronic GVHD (cGVHD) cases.^3^

#### Pathogenesis

Tissue damage activates host APCs which interact with donor T cells through CD80/CD86 and CD28 co-stimulatory molecules causing differentiation of donor T cells to Th1/Th17/Tc cell types, and eliciting an inflammatory cascade.^3^ Infiltrating immune cells release IFN-γ, TNF-α, and IL-2 posing cytotoxic effect on the target tissue.^3^ In the chronic stage, there is marked increase in numbers of macrophages and the allo/auto-reactive B cell population, along with an imbalance in the ratio of Treg and Th effector cells at the ocular surface.^9^ Overall, the ocular adnexa is damaged, which is characterized by fibrosis of the lacrimal gland (a hallmark of cGVHD), an impaired functioning of the meibomian glands (orifice hyperkeratinization, altered sebum quality, and cellular atrophy), eyelid inflammation, and persistent corneal and conjunctival epithelial defects (Figs. 1C, 1D).^9^^,^^10^

#### Clinical Diagnosis

Currently, the National Institutes of Health 2014 and International Chronic oGVHD consensus are the most adapted criteria for classifying oGVHD.^53^^,^^54^ Early detection of oGVHD is challenging due to our limited understanding of the underlying triggers, variable onset time relative to transplantation, and misdiagnosis due to overlapping symptoms (mucous discharge and crusting). In the chronic stage, patients with oGVHD are stricken with severe keratoconjunctivitis sicca, conjunctival congestion, irritation and pain in the eyes, foreign body sensation, redness, and vision blurring (see Figs. 1C, 1D).^9^^,^^10^

#### Ocular Surface Immune Dysregulation Reported At

##### Cellular Level

Inagaki et al. reported elevated macrophages at the corneal surface of patients with oGVHD (*n* = 4, on immunosuppression).^55^ Although T cells are known players in graft rejection, Eberwein et al. observed no difference in Tc levels in the conjunctiva of patients with oGVHD (*n* = 18, on medication) compared with HCs.^56^

Studies by Rojas et al., Razzaque et al., Tong et al., and Byun et al. compared immune cells pre- and post-transplantation and observed elevated macrophages, B cells, Tc cells, Th cells, and neutrophils, with no difference in NK cell levels in the conjunctiva.^57^^−^^60^ It is important to note that these observations are not based on a comparison with HCs. Figure 2A is a bubble graph overview of the reported immune cells at the ocular surface of oGVHD to date.

##### Molecular Level

Tears and conjunctiva of patients with oGVHD report elevated levels of IL-6, TGF-α/β, IFN-γ/α, and TNF-α. Irrespective of the patient's medication status, higher IL-6 levels were observed in tears (*n* = 20 and 34)^61^^–^^64^ and conjunctiva (*n* = 20)^65^ of oGVHD compared with HCs. Contrasting reports of IL-1β was observed, with a greater presence in conjunctival tissue (*n* = 20)^65^ and no difference in tears (*n* = 22)^66^ when compared with HCs in patients who were not on medication.

Because lacrimal gland fibrosis is a hallmark of oGVHD, the levels of most fibrosis-related factors (IL-13, TGF-β1, and IL-17; *n* = 20)^65^ when compared with HCs were not observed to be different in patients with oGVHD except for TGF-β2/3 (*n* = 20),^65^ which was reported to be higher compared with HCs.

Higher IFN-γ and TNF-α levels were observed in both tear (*n* = 34, 44, 24, and 20)^61^^,^^62^^,^^64^^,^^65^^,^^67^ and conjunctival samples (*n* = 20)^61^^,^^62^^,^^64^^,^^65^^,^^67^ of patients with oGVHD when compared with HCs. Nair et al. additionally observed a consistent elevation of IFN-γ levels for a year in oGVHD tears (*n* = 8).^64^ Although most of these studies had patients with oGVHD on immunosuppressive agents at the time of sample collection,^61^^,^^62^^,^^64^ Cocho et al. had patients discontinue medication for a week.^65^ Along with IFN-γ, Cocho et al. also reported higher IFN-α levels in the conjunctival region (*n* = 20).^65^

Similar to SJS, CXCL10 and CCL11 were also studied in oGVHD, with higher CCL11 (*n* = 20)^65^ levels reported in the conjunctiva and low CXCL10 levels (*n* = 22)^66^ reported in the tears (irrespective of medication status), when compared with HCs. Figure 2B is a bubble graph overview of the reported secretory factors at the ocular surface of oGVHD to date.

## Comparing the Cellular and Molecular Immune Changes at the Ocular Surface of OCP, SJS, and oGVHD

At the cellular level, only neutrophils have been studied in common, across all three conditions (OCP, SJS, and oGVHD; see Fig. 2A; Table 1). Among OCP and SJS, mast cells were reported to be elevated whereas monocytes were reported to be elevated only at the ocular surface of SJS although no difference was reported in the conjunctiva of patients with OCP (see Fig. 2A; Table 1). B cells, macrophages, NK cells, and Tc cells were commonly reported across OCP and oGVHD, with elevated B cells and macrophages reported in both the conditions (OCP and oGVHD), NK cells were reported to be elevated in oGVHD and no difference was observed in OCP, and Tc cells were reported to be elevated in oGVHD and contrasting reports (low and high) in the conjunctiva of OCP (see Fig. 2A; Table 1). Levels of DCs, NK T cells, eosinophils, and plasma cells have been studied only in OCP (see Table 1). Although all the cellular studies across these conditions have been done in conjunctiva, differences have been noted with respect to sampling techniques such as OSIC or tissue biopsies (see Table 1).

**Table 1.** Table Lists the Level of all Immune Cells Which Have Been Reported in all Three Conditions of OCP, SJS, and oGVHD

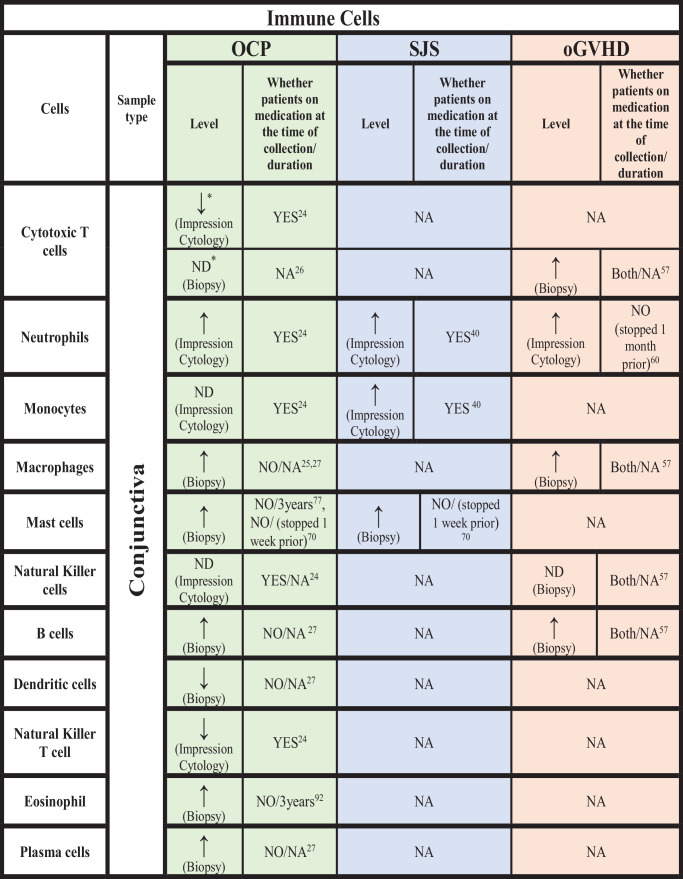

ND, no difference observed/reported for that specific cell; NA, data not available.

^*^ Highlighting the factors whose levels at the ocular surface show discrepancy.

At the molecular level, IL-10, IL-12α, IL-13, IL-6, IL-8, IFN-α, IFN-γ, and CXCL10 have been studied across all 3 OSIDs and reports note varied expression in each of the conditions as shown in Figure 2B and Tables 2, 3, and 4. Levels of other molecules like TNF-α, IL-1α in OCP; TNF-α, IL-10, CXCL10, IL-13, IL-12α, and IFN-γ in SJS; and MMP-9, CCL5, and CXCL10 in oGVHD have reported discrepancies (Figs. 4C, 4D, and 4E as red star mark). Two possible reasons proposed for the observed differences could be (1) the different ocular samples used for studying these secretory immune profile (see Figs. 4C–E; Tables 2, 3, 4) and (2) difference in patients’ medication status at the time of sample collection (Tables 2, 3, 4). Some of the key immune changes (cellular and molecular) observed in distinction and common reported across these OSIDs studies, which could be correlated to the clinical phenotypes (listed in Fig. 4A) has been depicted in Figure 4B. Studies are needed to address the research lacunae in terms of cells and factors that have not been studied to date in each of these OSIDs, which could be observed as gaps in Figures 2A and 2B.

**Table 2.** Table Lists the Level of Pro-Inflammatory Factors Which Have Been Reported in all Three Conditions of OCP, SJS, and oGVHD

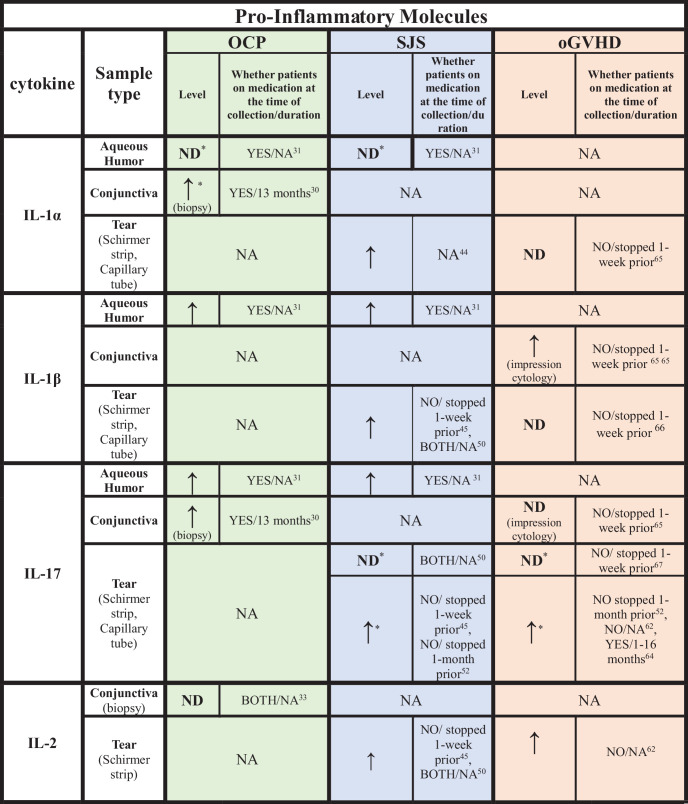
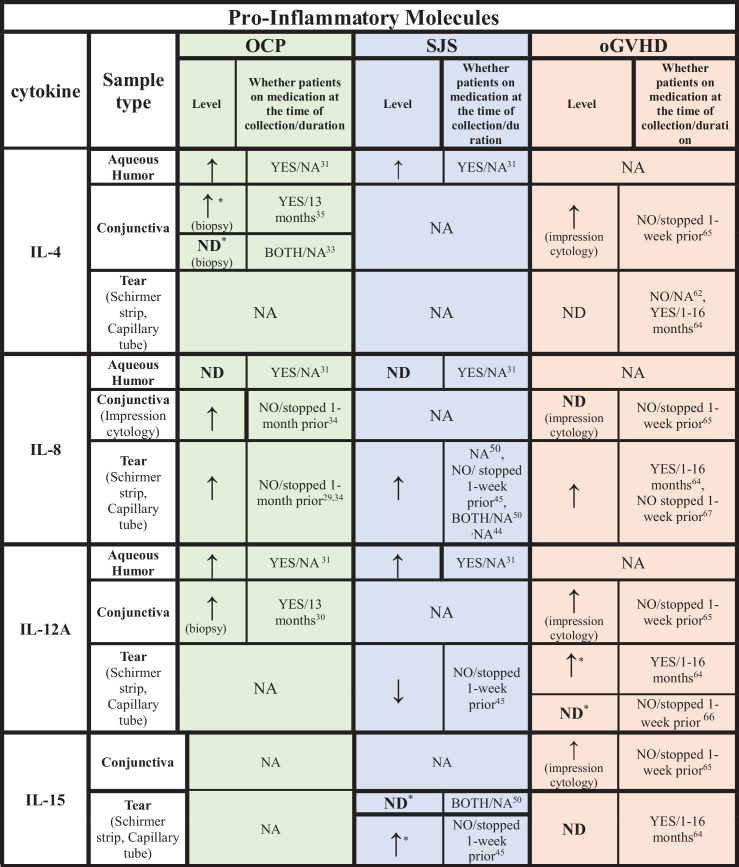
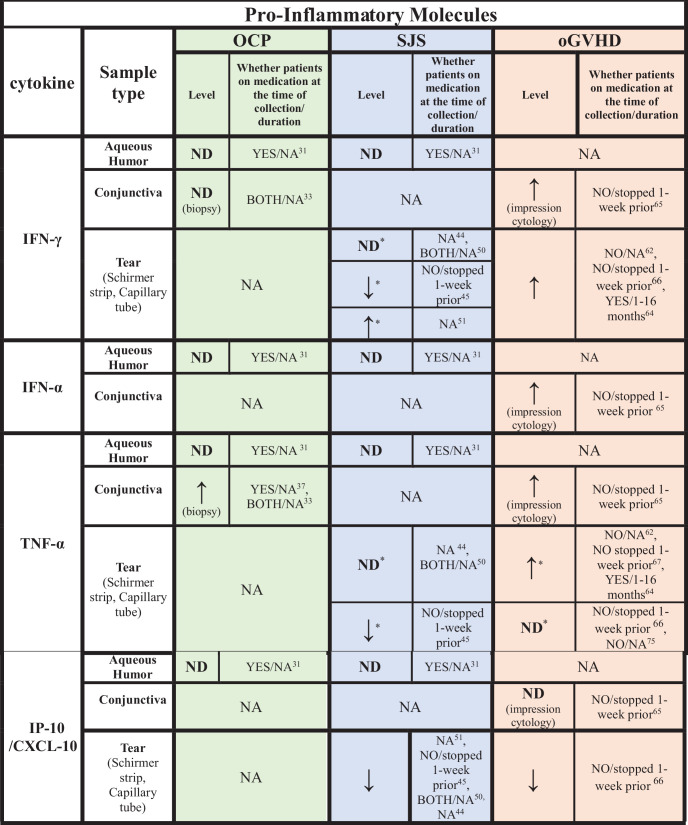
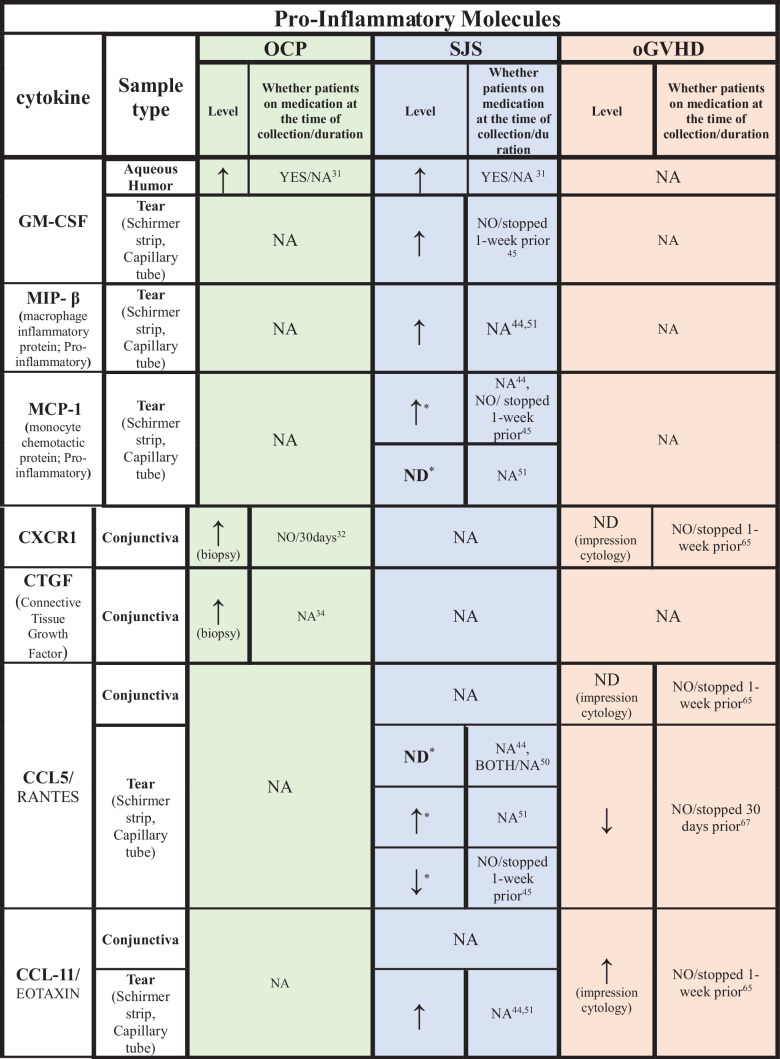

ND, no difference observed/reported for that specific cell; NA, data not available.

^*^ Highlighting the factors whose levels at the ocular surface show discrepancy.

**Table 3.** Table Lists the Level of all Anti-Inflammatory Factors Which Have Been Reported in all Three Conditions of OCP, SJS, and oGVHD

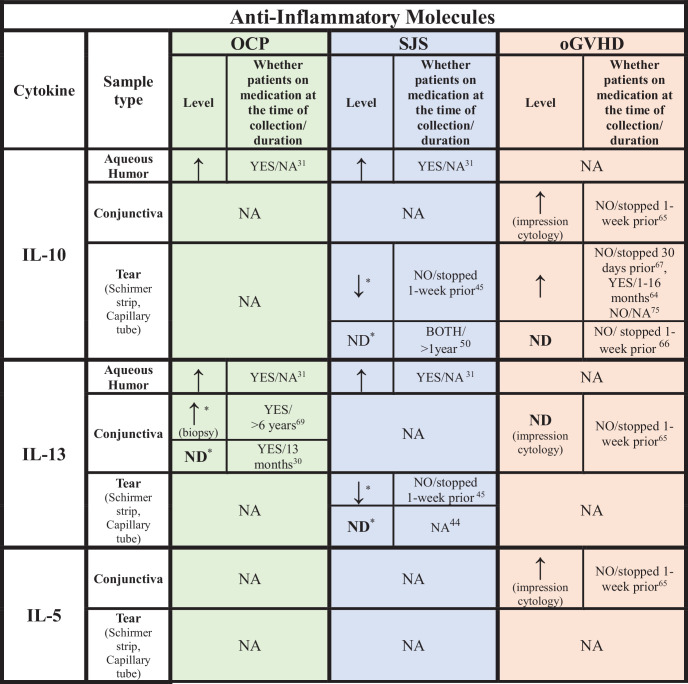

ND, no difference observed/reported for that specific cell; NA, data not available.

^*^ Highlighting the factors whose levels at the ocular surface show discrepancy.

**Table 4.** Table Lists the Level of all Secretory Factors Having Both Pro- and Anti-Inflammatory Properties Which Have Been Reported in all Three Conditions of OCP, SJS, and oGVHD

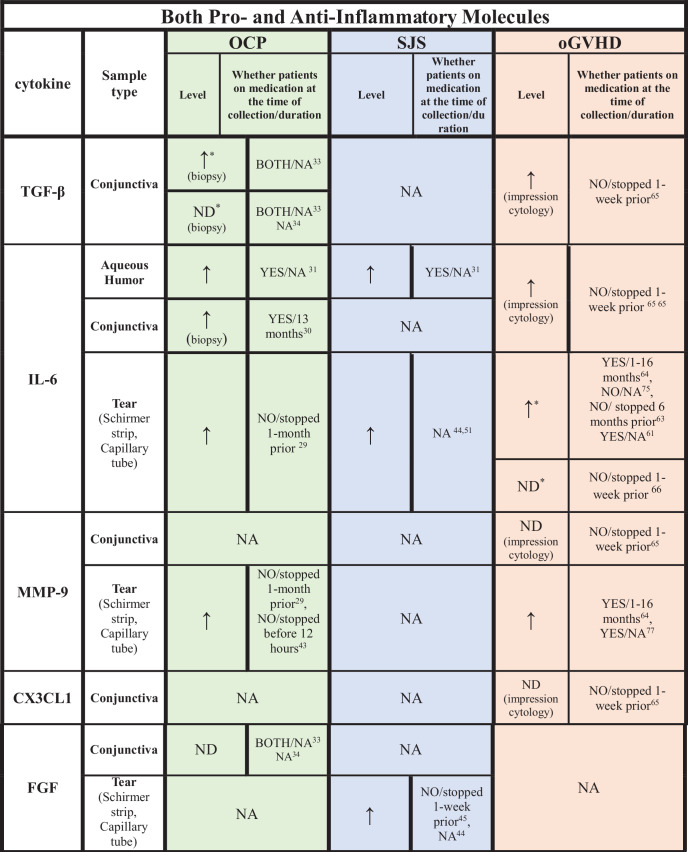
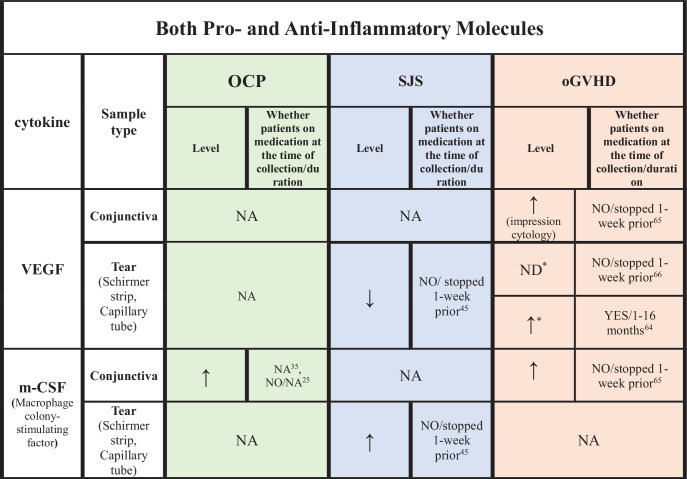

^*^Highlighting the factors whose levels at the ocular surface show discrepancy.

## Proposed Immunopathogenesis for the Focused OSIDs

### Proposed Immunopathogenesis for Ocular Cicatricial Pemphigoid

**Figure 5.**
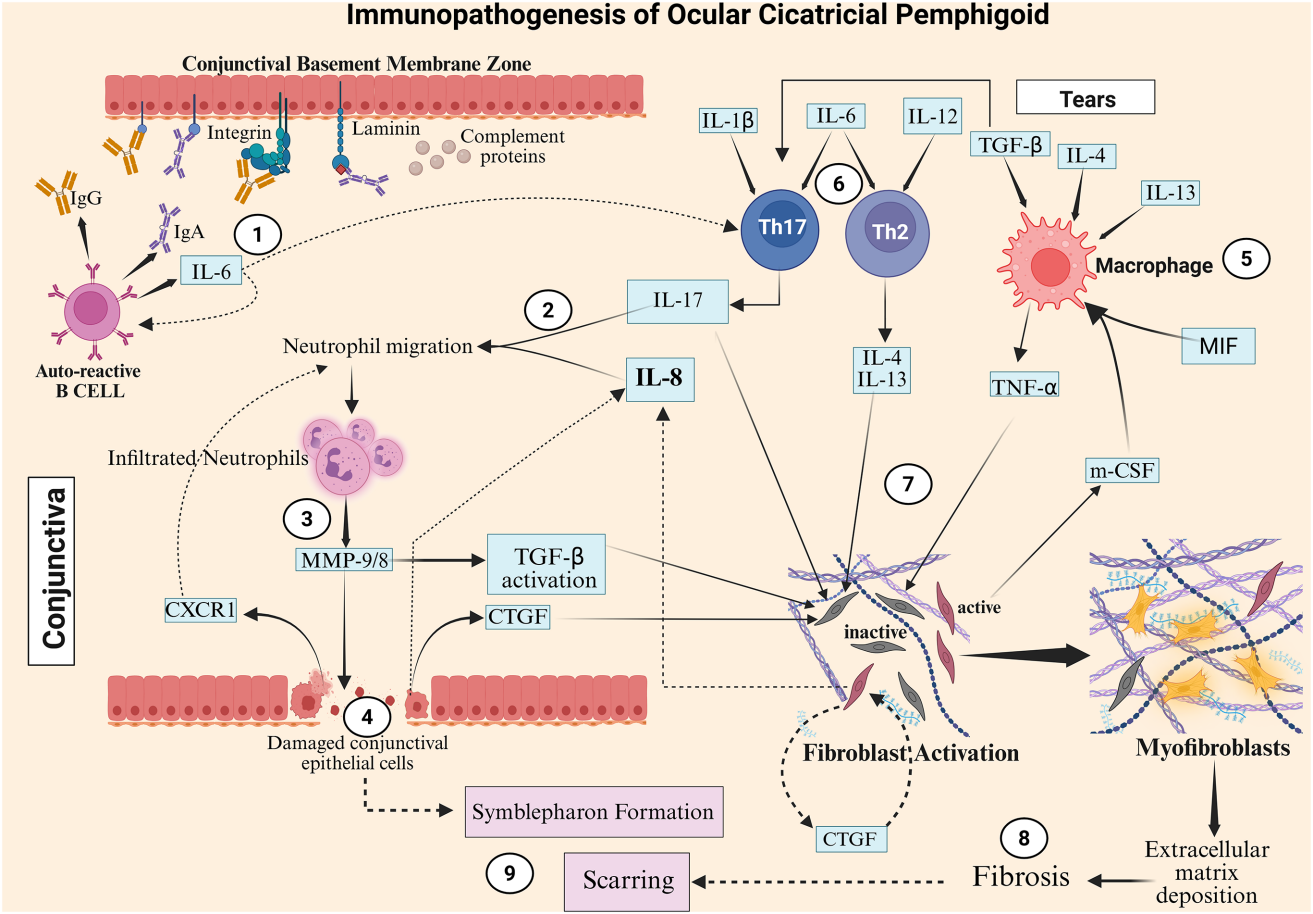
**Proposed immunopathogenesis of** **ocular** **cicatricial** **pemphigoid**. This is a schematic representation of pathophysiology proposed at the ocular surface of OCP condition based on the findings from the existing literature. (**1**) Autoreactive B cells could be maintained by high levels of tear IL-6 which could also activate Th2 and Th17 cells. (**2**) High levels of IL-17 from the activated Th17 cells along with IL-8 could elevate ocular neutrophil migration. (**3**) These neutrophils could activate MMP-9 and MMP-8 that in turn perform dual role by activating TGF-β also causing ECM degradation and damaging the conjunctival epithelial tissue. (**4**) The damaged epithelial cells could be one of the sources for IL-8, CXCR1 which could further increase ocular neutrophil levels and CTGF which could contribute to ocular surface fibrosis. (**5**) TGF-β along with Th2 derived cytokines (IL-4 and IL-13), MIF, and m-CSF could recruit and activate macrophages. (**6**) Other elevated pro-inflammatory cytokines, like IL-12, IL-6, and IL-1β, could further cause elevation of Th cells (Th2 and Th17). (**7**) Secretions from macrophages and Th cells, like TNF-α, IL-4, IL-13, and IL-17, could further contribute in activating fibroblast to myofibroblast transition. (**8**) The active myofibroblasts could cause irregular ECM deposition and contribute to fibrosis/scarring. (**9**) The active fibrosis and continuous damage to conjunctival epithelium could be the underlying factors behind key clinical phenotypes of OCP like symblepharon formation and conjunctival/corneal scarring (image created in https://BioRender.com).

OCP is currently categorized as a type II hypersensitive reaction, with B cells playing a primary role in its pathogenesis. They seem to be activated by unknown triggers to produce self-reacting antibodies (IgG/IgA/IgM) targeting conjunctival basement membrane proteins. Based on the existing literature, the immune cascade unfolding at the ocular surface could be proposed as follows and depicted in Figure 5.

(1) High levels of tear IL-6,^29^^–^^31^ a pleotropic cytokine which could be released under stress from various sources like fibroblasts (underlying the scarring), and by immune cells like macrophages, DCs, T (Th17) cells, and B cells. Having an autocrine function in B cells, IL-6 could be majorly contributing to maintaining higher levels of auto-reactive B cells while also maintaining the levels of Th2 and Th17 cells at the ocular surface.^26^ (2) Activated Th17, could produce one of its key cytokine IL-17^30^^,^^31^ which, along with IL-8, could further recruit neutrophils to the ocular surface.^24^ (3) Infiltrating neutrophils along with macrophages, activated fibroblasts, and the stressed epithelial cells could together activate MMP-9^29^^,^^43^ and MMP-8,^43^ which in turn plays a role in TGF-β activation^33^^,^^34^ and degradation of extracellular matrix (ECM) amplifying fibrosis, poor wound healing, and damaging the epithelial tissues (conjunctival/corneal) contribute to tissue scarring. (4) These infiltrating immune regulators and damaged epithelial cells at the ocular surface could enhance the production of CXCL8/IL-8,^29^^,^^34^ CXCR1,^32^ and CTGF.^34^ IL-8 and CXCR1 along with IL-17 to further go on to recruit and increase the ocular neutrophil percentages.^24^^,^^40^ (5) The activated TGF-β with IL-4,^35^^,^^68^ MIF,^58^ and IL-13^35^^,^^69^ (majorly secreted by activated Th2 cells) could recruit and activate macrophages.^25^^,^^27^^,^^70^ (6) Parallelly, other pro-inflammatory cytokines secreted at the ocular surface, like IL-12, IL-6, and IL-1β, could further activate Th cell types (Th2 and Th17). (7) Macrophages and Th cells in conjunction with stressed ocular epithelial cells could further amplify the signal to activate fibroblasts through cytokines like TNF-α, IL-4, IL-13, and IL-17.^33^^,^^37^ (8) These activated fibroblasts would differentiate to myofibroblasts, cause irregular ECM deposition, and contribute to the scarring of the cornea and conjunctiva. (9) The active fibrosis and damaged epithelia with high inflammation could result in the key clinical phenotypes of OCP, including symblepharon formation or conjunctival/corneal scarring.

### Proposed Immunopathogenesis for Stevens-Johnsons Syndrome

**Figure 6.**
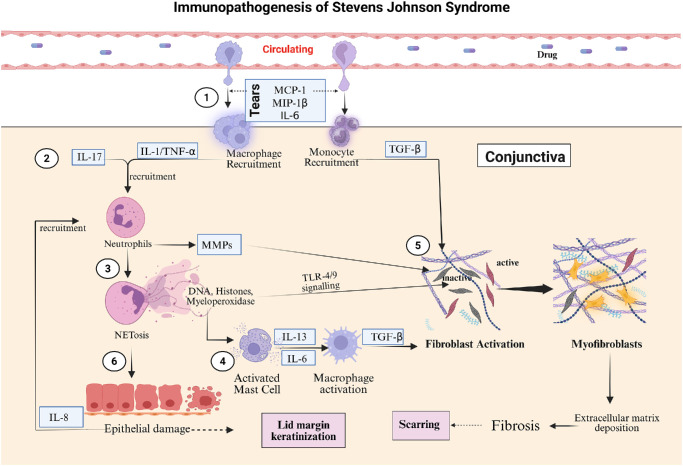
**Proposed immunopathogenesis of Stevens Johnson** **syndrome (SJS)**. This is a schematic representation of pathophysiology proposed for chronic ocular SJS based on the findings from the existing literature. (**1**) Post drug mediated systemic immune reaction, triggers (unknown) could initiate an inflammatory cascade in the eye leading to increased production of chemokines, like MCP-1/CCL2, IL-6, and MIP-1β/CCL4, which could recruit macrophages and monocytes to the ocular surface. (**2**) Pro-inflammatory cytokines released by the activated macrophages/monocytes, like IL-1 and TNF-α, along with other elevated tear cytokines, like IL-8 and IL-17, could enhance the recruitment of neutrophils to the ocular surface. (**3**) Inflammatory environment could drive these neutrophils to undergo NETosis, releasing intracellular in the form of NETs which is majorly composed of DNA, histone proteins, MPO, neutrophil elastase, and cathepsin G. (**4**) NETs have reported to have a role in aggravating inflammation, which could be through activation of mast cells or fibroblast cells. The activated mast cells could release IL-6 and IL-13 to activate macrophages which could also activate fibroblasts. (**5**) MMPs activated by neutrophils, TGF-β, and NETs together could further promote fibroblast to myofibroblast transition leading to irregular ECM deposition and scarring of cornea/conjunctiva. (6) NETs along with other inflammatory cascade could contribute in damaging the epithelial cells which could result in eyelid margin keratinization (one of the key clinical features) in the chronic SJS condition. Image created in https://BioRender.com.

It is well established that SJS is a drug-induced inflammatory response. It has a systemic phase marked by mucocutaneous blister formation and epidermal surface detachment, majorly attributed to Tc cells. However, the immune mechanism underlying the ocular surface damage remains unexplored. From the existing literature, the immune cascade unfolding at the ocular surface immune could be proposed as depicted in Figure 6.

(1) An unknown stimulus following the systemic drug reaction could initiate the inflammatory cascade at the ocular surface. Ocular cells, like epithelial cells, fibroblasts, and resident immune cells (macrophages or DCs), under stress could release various inflammatory molecules like MCP-1/CCL2, (Aketa et al., 2017; Gurumurthy et al., 2018b; Yoshikawa et al., 2020) IL-6, and MIP-1β/CCL4,^31^^,^^44^^,^^51^ which could in turn enhance the levels of macrophages and monocytes at the ocular surface. (2) These activated macrophages and monocytes along with damage-associated molecular patterns (DAMPs) could contribute to the release of various pro-inflammatory cytokines like IL-1^44^^,^^50^ and TNF-α,^71^ which along with IL-8 (Gurumurthy et al., 2018; Koduri et al., 2021a, 2024; Ueta et al., 2017; Yoshikawa et al., 2020) and IL-17 (Aketa et al., 2017; Gurumurthy et al., 2018b; Kang et al., 2011) could further recruit and increase the number of neutrophils at the ocular surface.^40^^,^^41^^,^^46^ (3) These neutrophils (due to unknown triggers) could undergo programmed cell death (NETosis), which causes the release of neutrophil DNA into the extracellular environment termed neutrophil extracellular traps (NETs). The NETs are composed of nucleic acids, histone proteins, and lytic enzymes like myeloperoxidase (MPO), neutrophil elastase, or cathepsin G.^41^ (4) NETs could in turn play a role as autoantigens and aggravating the inflammatory response, including activation being of the mast cells,^70^ that could release IL-6 and IL-13^31^^,^^71^ which in turn could activate macrophages.^46^^,^^48^ (5) TGF-β from various sources like damaged epithelial cells, fibroblasts, and macrophages/monocytes along with MMPs and MPO^46^ could further amplify fibrosis at the ocular surface. (6) NETs along with the infiltrated immune cells and factors along with the signals from a stressed epithelium could contribute in damaging the surface epithelium (conjunctival/corneal tissues) thereby becoming one of the underlying causes for key clinical features of SJS such as eyelid margin keratinization.

### Proposed Immunopathogenesis for Ocular Graft Versus Host Disease

**Figure 7.**
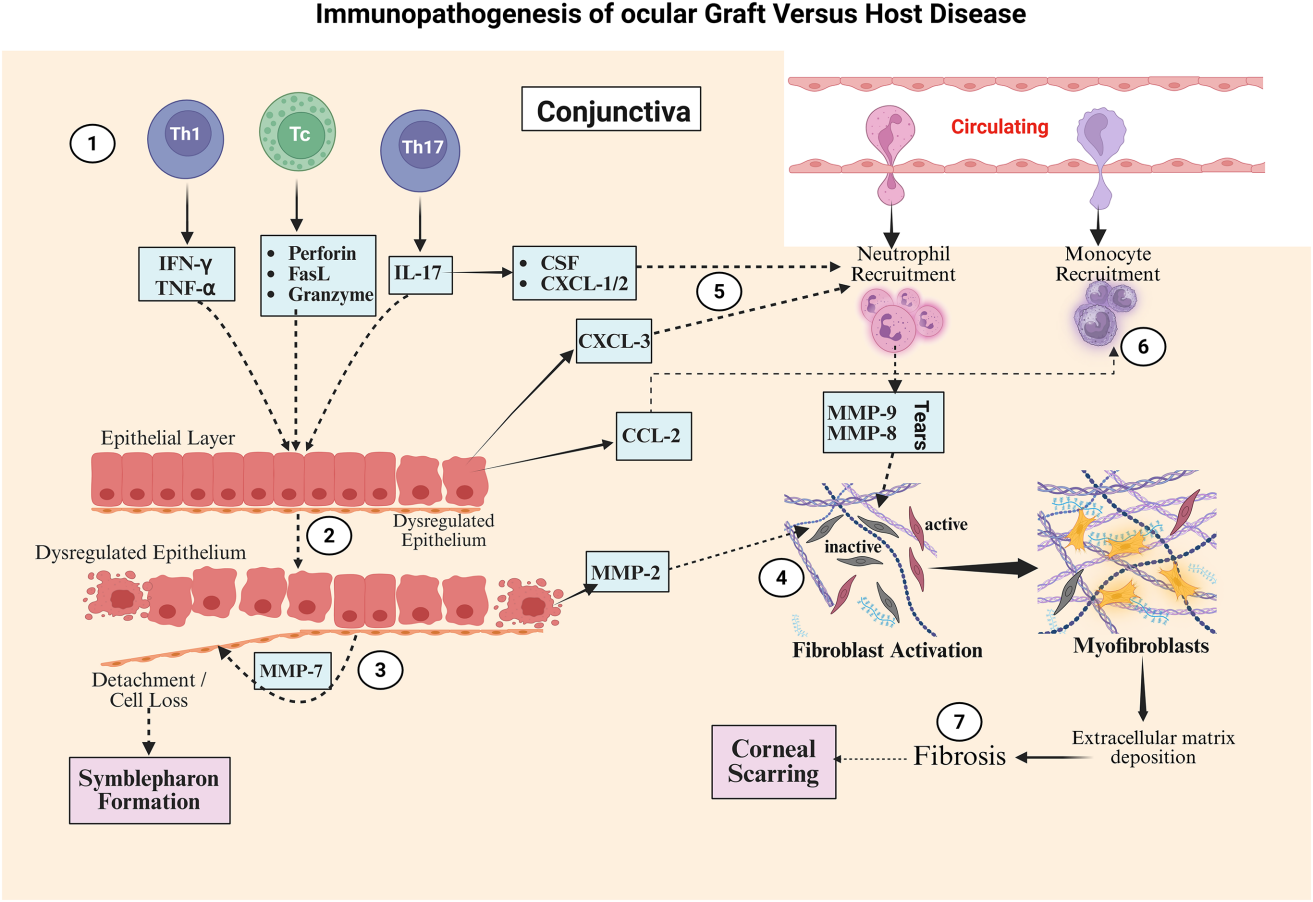
**Proposed immunopathogenesis of ocular** **graft** **versus** **host** **disease (oGVHD).** This is a schematic representation of the proposed immunopathological changes occurring at the ocular surface of oGVHD individuals based on the finding from the existing literature. (**1**) Post hematopoietic stem cell transplantation, the immune system of the individual is compromised leading to infiltration of donor T cells into various target tissue including the eye. These T cells, both Th cells (Th1 and Th17) and Tc cells could release various inflammatory factors like IFN-γ, TNF-α, IL-17, perforins, Fas ligand, and granzymes which could contribute to (**2**) epithelial layer deterioration, in the process releasing several chemokines and factors to activate ocular MMPs. These include (**3**) MMP-7, which could further amplify the epithelial damage and contribute to conjunctival symblepharon formation, and (**4**) MMP-2 which could activate ocular fibroblasts along with MMP-9/8 (released by ocular neutrophils). (**5**) Chemokines CXCL3/MIP-2β from damaged epithelial cells in synergy with Th17 associated factors IL-17, CSF, and CXCL-1/2 could further enhance neutrophils migration to the ocular surface. (**6**) Another chemokine CCL2 released from damaged epithelium could cause migration of monocytes to the ocular surface. (**7**) The infiltrated milieu could contribute ECM damage and lead to conjunctival/corneal scarring. Image created in https://BioRender.com.

The systemic manifestations of GVHD are primarily driven by T cells (both helper and cytotoxic). Researchers have reported elevation of T cells even in the lacrimal gland. Based on current evidence, the immunopathogenesis occurring at the ocular surface of oGVHD could be proposed as (Fig. 7^8^): (1) infiltrating Th cells (Th1 and Th17)^57^^,^^59^^,^^72^^–^^74^ and Tc cells^57^^,^^72^^–^^74^ could be the source for various inflammatory molecules like IFN-γ,^61^^,^^62^^,^^64^^–^^66^^,^^75^ TNF-α,^64^^,^^65^^,^^67^ and IL-17,^52^^,^^64^^,^^65^ which in turn cause (2) epithelial deterioration. (3) Inflammation-induced elevation of various MMPs, like MMP-7^64^^,^^76^ and MMP-2,^76^ could also contribute to impaired epithelial function, where MMP-7 primarily damages the epithelium and (4) MMP-2, along with MMP-9^64^^,^^65^^,^^76^^,^^77^ and MMP-8,^76^ causes fibroblast activation. (5) The damaged epithelium could further release CXCL3/MIP-2β^65^ which along with IL-17 induced CXCL1/2^65^ and CSF,^65^ could promote neutrophil recruitment^60^ to the ocular surface. (6) CCL2,^65^ another chemokine released by the damaged epithelium, could further enhance recruitment of monocytes to the ocular surface.^57^ (7) Overall, the immune disbalance could lead to fibrosis at the ocular surface (see Fig. 7).

**Figure 8.**
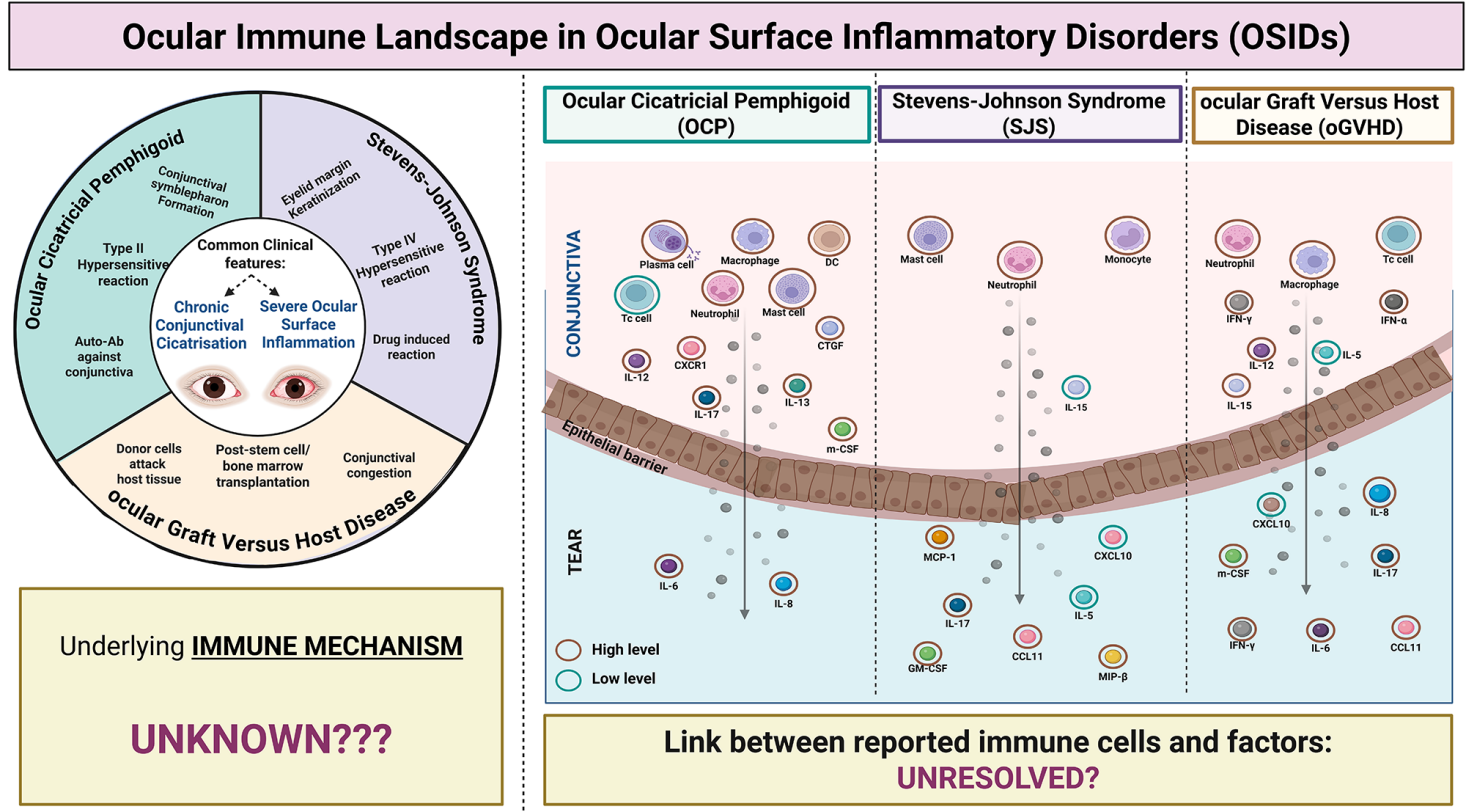
**Ocular immune landscape in Ocular Surface Inflammatory Disorders.** OSIDs such as OCP, SJS, and oGVHD stem from different etiologies but share chronic conjunctival cicatrizing inflammation along with limited knowledge of the underlying immune mechanism. Review highlights the potential role of several cellular and molecular immune players reported across the studies while underscoring the need to establish correlation among these factors for improving targeted therapies.

## Research Challenges and Future Perspectives

Based on our current immunological understanding of the focused OSIDs, the following research lacunae can be highlighted:

• Studies have reported active clinical phenotypes in eyes that appear without inflammation,^24^^,^^40^ but minimal understanding exists about the immunological differences between inflamed and non-inflamed cases.
• In OCP, limited studies have been conducted to understand the stimuli/trigger underlying the loss of self-tolerance in B cells, leading to autoantibody production and ocular tissue damage.
• A systemic phase of SJS is evidently reported to be driven by T cells (especially Tc cells), but ocular status of T cells remains unexplored in SJS.
• Regulatory T cells, one of the key immune regulators, have not been studied at the ocular status of these OSIDs.
• A research gap exists in our understanding of the ocular surface levels of several immune cells and secretory factors for each OSID groups, as observed in Tables 1 to 4 (cells having NA/not available) and blank spaces in Figures 2A and 2B.
• Across these groups, patients are treated with immunosuppressants, which could alter immune status locally/systemically, as observed in Tables 1 to 4. Future studies could rule out the medication effect on the ocular immune profile.
• Another major technical lacuna observed was different sampling techniques used for local immune profiling (as observed in Figs. 4A–E), which suggest the need for a uniform, sensitive, and reproducible sample collection method to better define the ocular immune landscape in these conditions.

## Summary

We report and compare the immunological differences at the ocular surface of three OSIDs: SJS, OCP, and oGVHD. Poorly defined immune factors underlying their ocular phenotypes, limit the targeted therapies for these conditions. An overview of the current reported immunological changes across these OSIDs has been summarised in Figure 8.

Neutrophils have been reported to be elevated in the conjunctiva of these OSIDs and could be correlated to increased IL-8, neutrophil elastase, and MPO levels locally. Macrophages have been elevated in the conjunctiva of both OCP and oGVHD with pro-inflammatory associated proteins elevated in oGVHD (IL-6, TNF-α, and IL-1β) and anti-inflammatory associated proteins elevated in OCP (TGF-β1 and IL-10; see Tables 2, 3; Fig. 2B). In the conjunctiva of SJS, monocytes and mast cells were also elevated (see Table 1; Fig. 2B), which can be corroborated with elevated factors like MCP-1, IL-6, IL-1β, GM-CSF and MIP-β/CCL4, and CCL5 (see Table 2; Fig. 2B). Conjunctival Tc cells were reported lower in OCP, higher in oGVHD, and unexplored in SJS. Tc associated factors were correlated in only oGVHD with elevation in IFN-γ, TNF-α, IL-2, and IL-12 (see Table 2; Fig. 2B). Elevated levels of Th1-associated factors (IFN-γ, IL-2, TNF-α, and IL-12; see Table 2; Fig. 2B) in both SJS and oGVHD and Th17-associated factors (IL-17A; see Table 2; Fig. 3B) in oGVHD ocular surface indicates the role of helper T cells in these conditions. Local elevation of CCL11/eotaxin and CCL5 (see Table 2; Fig. 2B), two of the major eosinophil chemokines in SJS indicates eosinophil involvement.

On comparing the data from many studies, a discrepancy in the levels of various cells and secretory factors could be observed at the ocular surface (highlighted as “*” in Tables 1^2^,^3^–4) For instance, levels of Tc cells, IL-1α, IL-4, IL-13, and TGF-β in the conjunctiva of patients with OCP (see Tables 1^2^,^3^–4). Similar observations were made for the tear levels of IL-17, IL-15, IFN-γ, TNF-α, MCP-1, CCL-5, IL-10, and IL-13 in patients with SJS and IL-17, IL-12, TNF-α, IL-6, and VEGF in patients with oGVHD, respectively (see Tables 2^3^–4). One of the reasons for this cellular/molecular discrepancy could be due to different sampling types (conjunctival tissue versus biopsy or tear Schirmer versus capillary tube). To address this issue, there is a need to standardize the sampling protocol for assessing the changes in inflammatory cells and factors at the ocular surface. Our group had highlighted the importance of buffer choice used for tear elution from the Schirmer strip on the total protein yield.^78^ Recently, Gijs and group^79^^,^^80^ have been working toward this technical lacuna by comparing the efficacy of tear collection methods mentioned in literature to date. The comparison is being made in various aspects (from the time of collection, buffer used for eluting tears, and incubation time to storage temperature), with an aim to standardize the sampling technique for better assessment of tear biomarkers at the global level.^79^^,^^80^ In considering the importance of this lacunae, future studies should compare the inflammatory markers (cellular and molecular) in different adapted sampling techniques for better understanding of the immune mechanisms of cicatrizing ocular surface diseases. Our group is also working toward using a noninvasive, uniform ocular surface wash samples to understand and compare ocular immune profile across SJS, OCP, and oGVHD. Another reason for these immune variations could be the differences in patients’ medication status at the time of sample collection. Because immunosuppressants are known to alter immune status the studies should rule out the effect of immunosuppressive agents for better immune profile comprehension.

Ocular surface (cornea, conjunctiva, and limbus) is populated with resident immune cells, namely CCR2± macrophages,^18^ CD11c+CD11b-/low Langerhans DCs, CD11c+CD11b+CD207+ non-Langerhans DCs,^15^^,^^17^ γδ T cells,^18^^,^^19^ CD8+ ^effector^ memory T cells, and CD8+ effector memory T cells.^81^ From the current findings, the levels of these resident immune cells apart from macrophages (in OCP^25^^,^^27^^,^^58^ and oGVHD^57^) has not been evaluated at the ocular surface. Along with the infiltrating immune cells, it is important to understand the status of these resident immune cells across these OSIDs. Apart from harboring immune cells, the cornea is one of the highly innervated parts of the eye. Studies have shown the role of neuro-immune crosstalk in driving the fate of inflammation of various systemic and ocular conditions including dry eye, rheumatoid arthritis, glaucoma, and diabetes mellitus.^82^^,^^83^ Dry eye condition remains the most reported ocular co-morbidities in the focused OSIDs (OCP, SJS, and oGVHD). Studies have shown neuropeptides, like substance P, can elevate the corneal levels of Th cells and switching the memory T cells (one of the resident immune cells) to Th17 type in desiccation-induced dry eye animal models, thereby amplifying the inflammation.^82^^,^^83^ In corneal epithelial injury models, elevated neutrophils have a beneficial effect on corneal nerve regeneration, whereas elevated corneal DCs correlate to ocular discomfort.^82^^,^^83^ Based on these findings, to associate the underlying cause of chronic ocular damage in these OSIDs, the ocular levels of infiltrating and resident immune cells (cornea and conjunctiva) should be evaluated along with neuropeptides (substance P, calcitonin gene-related peptide, nerve growth factor, or other neurotrophins) to understand the effect of neuro-immune crosstalk in the pathology of these cicatrizing conditions. Another key clinical feature reported in common across these OSIDs (OCP, SJS, and oGVHD) is fibrosis, where the dysregulated fibroblasts produce irregular ECM and cause scarring. The role of TGF-β is well established in maintaining subepithelial/stromal myofibroblasts whereas IL-1α/β signals apoptosis of myofibroblast.^84^^–^^87^ In normal conditions, corneal fibroblasts, keratocytes, and the immune cells release IL-1α/β, which through paracrine action, regulate the myofibroblasts.^84^^–^^87^ Assessing the ratio of TGF-β and IL-1 at the ocular surface of these OSIDs could enhance the understanding of the fibrosis. It is important to remember that the effect of infiltrating immune cells in these chronic inflammatory conditions will not be standalone, rather a crosstalk among the neurons, ocular cells (epithelial and stromal), fibroblasts, and resident and migrating immune cells, which will overall define the underlying pathology and the approach for targeted therapeutics. Based on the reported studies where elevated levels of IL-6, IL-1β, and IL-17 have been widely reported at the ocular surface, the existing US Food and Drug Administration (FDA) approved biologics, such as Anakinra/Kineret^88^ (IL-1β inhibitor for an autoimmune condition rheumatoid arthritis), Tocilizumab^89^ (IL-6 inhibitor for autoimmune condition systemic juvenile idiopathic arthritis), and Secukinumab, Brodalumab, and Ixekizumab^90^ (IL-17 inhibitors for autoimmune condition psoriasis) could be considered for repurposing. At the ocular surface however, Adalimumab^91^ (TNF-α inhibitor), remains the only FDA approved biologic used for uveitis. These biologics could be repurposed for topical or subconjunctival therapy in these chronic inflammatory ocular surface diseases. Exploring these therapies in the animal models of ocular surface damage would be the next right approach before clinical trials. The long-term goal would be modulating the identified immunological targets to alleviate the ocular inflammation and improve the quality of life.

## Methodology

This narrative review is based on our own research experience, previously published work, and clinical practice in one of the Asia's largest clinical settings in ophthalmic research. A literature survey was performed in two databases (PubMed and Google Scholar), with a search for keywords like “ocular AND pemphigoid” for OCP, “ocular AND graft versus host disease” for oGVHD, and “ocular AND Stevens-Johnson Syndrome” for SJS, respectively, over the last 30-year period. The results were primarily filtered for “research articles” followed by searching for terms “immune” in these research articles, to search for the abstracts that discuss the role of immune factors (cellular and molecular) relevant for each condition (OCP, SJS, and oGVHD). This resulted in a total of 67 papers in OCP, 74 papers in SJS, and 57 papers in oGVHD. The content in these filtered research articles were carefully categorized into several parameters as mentioned below for further screening to finally summarize the findings from 29 research articles from OCP, 27 from SJS, and 29 from oGVHD. These parameters included: (a) model type used in the study (human/animal/in vitro), only studies with human subjects were chosen for the review while excluding the case studies. (b) Disease stage, both acute and chronic case-based studies have been incorporated in the review. (c) Region of sample manifestation (conjunctiva/skin/blister fluid/aqueous humor/blood/serum), with the research articles where ocular samples were used for study design (conjunctiva/aqueous humor/tears) were chosen for this review, because our prime focus was to compare the ocular immune profile across these conditions. (d) Technique used to collect the ocular samples (biopsy/ Schirmer's strip/capillary tube/ocular surface impression cytology/surgery-based intervention) were noted down to understand the effect of sampling techniques in identifying differences in immunological changes. (e) Type of immune factor analyzed (cell/secretory factor) was another key inclusion. (f) In addition, the type of control group (healthy/disease control) used for comparison was important, and only studies where healthy controls were used to compare the immune factors were included in the study. (g) Finally, the patients’ medication status at the time of ocular sample collection was another key criterion, as these patients (with OCP, SJS, and oGVHD) are very likely on immunosuppressants, which could alter the ocular immune profile. It is important to note that we did not adhere to the guidelines for a systemic review, and the absence of risk-of-bias assessment constitutes a key methodological limitation. Overall, this narrative review summarizes the findings from the research articles focusing on human subjects (both acute and chronic stage), where the ocular surface was the target region of interest. The data (both cellular and molecular) was compared against healthy controls, while making a note of the sampling technique used and status of their medication at the time of sample collection.
